# Upfront systemic chemotherapy and preoperative short-course radiotherapy with delayed surgery for locally advanced rectal cancer with distant metastases

**DOI:** 10.1186/1748-717X-6-99

**Published:** 2011-08-24

**Authors:** Sang Joon Shin, Hong In Yoon, Nam Kyu Kim, Kang Young Lee, Byung Soh Min, Joong Bae Ahn, Ki Chang Keum, Woong Sub Koom

**Affiliations:** 1 Department of Medical Oncology, Yonsei Colorectal Cancer Clinic, Yonsei University College of Medicine, Seoul, Korea; 2 Department of Radiation Oncology, Yonsei Colorectal Cancer Clinic, Yonsei University College of Medicine, Seoul, Korea; 3 Department of Surgery, Yonsei Colorectal Cancer Clinic, Yonsei University College of Medicine, Seoul, Korea

**Keywords:** short-course radiotherapy, delayed surgery, locally advanced rectal cancer, distant metastases

## Abstract

**Background:**

Choosing the most effective approach for treating rectal cancer with mesorectal fascia (MRF) involvement or closeness and synchronous distant metastases is a current clinical challenge. The aim of this retrospective study was to determine if upfront systemic chemotherapy and short-course radiotherapy (RT) with delayed surgery enables R0 resection.

**Methods:**

Between March 2009 and October 2009, six patients were selected for upfront chemotherapy and short-course RT (5 × 5 Gy) with delayed surgery. The patients had locally advanced primary tumors with MRF involvement or closeness, as well as synchronous and potentially resectable distant metastases. Chemotherapy was administered to five patients between the end of the RT and surgery. All patients underwent total mesorectal excision (TME).

**Results:**

The median patient age was 54 years (range 39-63). All primary and metastatic lesions were resected simultaneously. The median duration between short-course RT and surgery was 13 weeks (range, 7-18). R0 resection of rectal lesions was achieved in 5 patients. One patient, who had a very low-lying tumor, had an R1 resection. The median follow-up duration for all patients was 16.7 months (range, 15.5-23.5). One patient developed liver metastasis at 15.7 months. There have been no local recurrences or deaths.

**Conclusions:**

Upfront chemotherapy and short course RT with delayed surgery is a valuable alternative treatment approach for patients with MRF involvement or closeness of rectal cancer with distant metastases.

## Background

Preoperative short-course radiotherapy (RT) or chemoradiotherapy followed by total mesorectal excision (TME) is an established treatment regimen for stage II and III rectal cancer [1-4]. In addition, the mesorectal fascia (MRF) involvement is known to predict the probability for local tumor recurrence and patient survival [5-7]. High resolution magnetic resonance imaging (MRI) can reliably and accurately assess the MRF involvement of rectal masses [8-10]. A Dutch TME trial showed that preoperative short-course RT does not compensate for positive circumferential resection margins (CRM) [11,12]. Therefore, when either MRF involvement or closeness is identified by MRI, patients require a treatment strategy that induces tumor regression and thereby enables an uninvolved MRF after TME. Conventional long-course RT with chemotherapy followed by delayed surgery is widely accepted as the standard approach for this patient group [13].

It is a current and critical challenge to determine the most effective treatment regimen for patients with MRF involvement and potentially resectable synchronous distant metastases, which differ from widely-spread systemic disease. In this patient group, there is a risk of distant metastases arising during conventional long-course chemoradiotherapy, but also a risk of local progression in preoperative combination chemotherapy (with or without an antibody) regimen to target metastatic disease without pelvic RT. Radu *et al*. suggested that upfront combination chemotherapy and short-course RT followed by delayed surgery might be a useful alternative treatment option for patients with locally advanced, non-resectable (T4) rectal cancer and synchronous distant metastases [14]. However, clinical evidence to support this theory is lacking.

The aim of this retrospective study was to determine if upfront systemic chemotherapy and short-course RT with delayed surgery is an effective treatment regimen for patients with MRF involvement or closeness and synchronous distant metastases.

## Methods

### Patient selection

Between March 2009 and October 2009, six patients were selected for upfront chemotherapy and short-course RT with delayed surgery. The patients had locally advanced primary tumors with MRF involvement or closeness, as well as synchronous and potentially resectable distant metastases. All patients had biopsy-confirmed adenocarcinoma as the primary rectal lesion. Patients had an Eastern Cooperative Oncology Group performance scale grade of 0, normal pre-treatment blood counts, and renal and liver function tests. Data were collected through retrospective review of medical records.

### Multidisciplinary team approach

The assessments and treatment approaches were determined at a multidisciplinary team conference in the Yonsei Colorectal Cancer Clinic. The patients were identified as candidates for the upfront systemic chemotherapy and short-course RT with delayed surgery, with the intention to perform the R0 resection for TME after tumor regression and simultaneous complete resection of metastatic lesions. This conception was proposed by Kim NK. The resectability of rectal and metastatic lesions was determined by the surgeon and radiologist based on imaging studies. All patients received 4 to 9 cycles of the upfront systemic chemotherapy with a FOLFOX based regimen (5-FU/leucovorin/oxaliplatin combination) with or without Bevacizumab (Avastin) or Cetuximab (Erbitux). These patients received RT of 25 Gy in five fractions during 5 consecutive work days after upfront chemotherapy. The same chemotherapy regimen was maintained between the end of the RT and the surgery with the intention of allowing time for tumor regression. Chemotherapy administered in one week after RT. One patient did not receive chemotherapy during the regression period. The resection of the primary and metastatic lesions was performed after re-evaluation of tumor stage and resectability at least 6 weeks after RT.

### Radiotherapy

All patients underwent planning computed tomography (CT) in the treatment position (prone with a full bladder) on the belly board. The gross tumor volume (GTV) was defined as the primary tumor and any significant surrounding lymphadenopathy, including lateral lymph nodes. The clinical target volume (CTV) was defined as follows; (1) a margin of at least 2 cm in the superior and inferior directions was added to the GTV; (2) the lateral margin encompassed the entire mesorectum at the level of the GTV; (3) When there was significant lateral lymph node involvement, a 1 cm margin was added to the GTV in all directions. Elective nodal irradiation was not performed to minimize small bowel irradiation. RT planning was accomplished using a 5-field technique (anterior-posterior beam, right-lateral beam, right-posterior-oblique beam, left-posterior-oblique beam, left-lateral beam) to cover the CTV plus 1 cm margin. The CTV was covered within the 95% isodose line.

### Tumor assessment, follow up, and statistics

Pretreatment staging work up included digital rectal examination, sigmoidoscopy, pelvic CT or MRI to evaluate local tumor extent and the involvement of MRF, chest radiography, CT scan of chest and abdomen, and positron emission tomography to diagnose the distant metastasis. Imaging studies were repeated after completion of the radiotherapy to evaluate the response and the resectability of the rectal and metastatic lesions. Follow-up visits were recommended at 1, 3, 6, and 12 months after surgery. Follow-up imaging studies were performed at 1, 6, and 12 months after surgery, and treatment-related toxicities were evaluated at every follow-up visit. We evaluated acute toxicities between RT and surgery, which refer to acute toxicities. Also, surgical complication was evaluated. Toxicity was evaluated according to the Common Toxicity Criteria Version 3.0 from the National Cancer Institute (NCI-CTC v3.0). Data for six patients were analyzed using SPSS version 18.0 software (SPSS Inc., Chicago, IL, USA)

## Results

### Patient characteristics and treatment

Patient characteristics at diagnosis are shown in Table 1. The median age was 54 years (range 39-63 years). Four patients were male and two were female. The tumor was localized at the middle third for four patients, and the lower third for two patients. The median distance from the anal verge was 7 cm (range 1-8 cm). The tumor was classified as T3 for three patients and T4 for three patients. There was one patient with an N0 classification, two with an N1 classification, and three with an N2 classification. Five patients had MRF involvement, and one patient had MRF closeness. All patients were diagnosed with distant metastasis. There were five patients with liver metastasis, two with ovarian metastasis, and one with peritoneal metastasis. Two patients had multiple sites of distant metastasis (Patients 2 and 4). Systemic chemotherapy prior to short-course RT was given to all patients. All patients except Patient 4 were treated with three to five cycles of additional systemic chemotherapy during the interval between RT and surgery.

**Table 1:** Patient characteristics and treatments

**Patient No**.	Age (years)	Gender	Pathology	AV (cm)	Initial stage*	MRF involvement	DM site	Preoperative treatment
1	52	M	Adeno MD	7.8	T4aN1M1a	+	Liver	E-FOLFOX #9 + RT (25 Gy/5fx) + E-FOLFOX #3
2	39	F	Adeno MD	7	T4aN0M1b	+	Peritoneum, Lt. ovary	A-FOLFOX #7 + RT (25 Gy/5fx) + A-FOLFOX #2
3	56	M	Adeno MD	4	T3N2aM1a	+	Liver	FOLFOX #4 + RT (25 Gy/5fx) + FOLFOX #4
4	45	F	Adeno WD	8	T3N2aM1a	Threatened	Liver, both ovaries	FOLFOX #4 + RT (25 Gy/5fx)
5	63	M	Adeno MD	1	T4N1aM1a	+	Liver	FOLFOX #4 + RT (25 Gy/5fx) + FOLFOX #4
6	60	M	Adeno MD	7	T3N2bM1a	+	Liver	FOLFOX #4 + RT (25 Gy/5fx) + FOLFOX #5

*Abbreviations: *AV, anal verge; MRF, mesorectal fascia; adeno, adenocarcinoma; MD, moderately differentiated; Lt., left; FOLFOX, 5-fluorouracil/leucovorin/oxaliplatin; E, erbitux; RT, radiation therapy; A, avastin.

*AJCC 7^th ^edition.

### Treatment response and surgery

**Figure 1:**
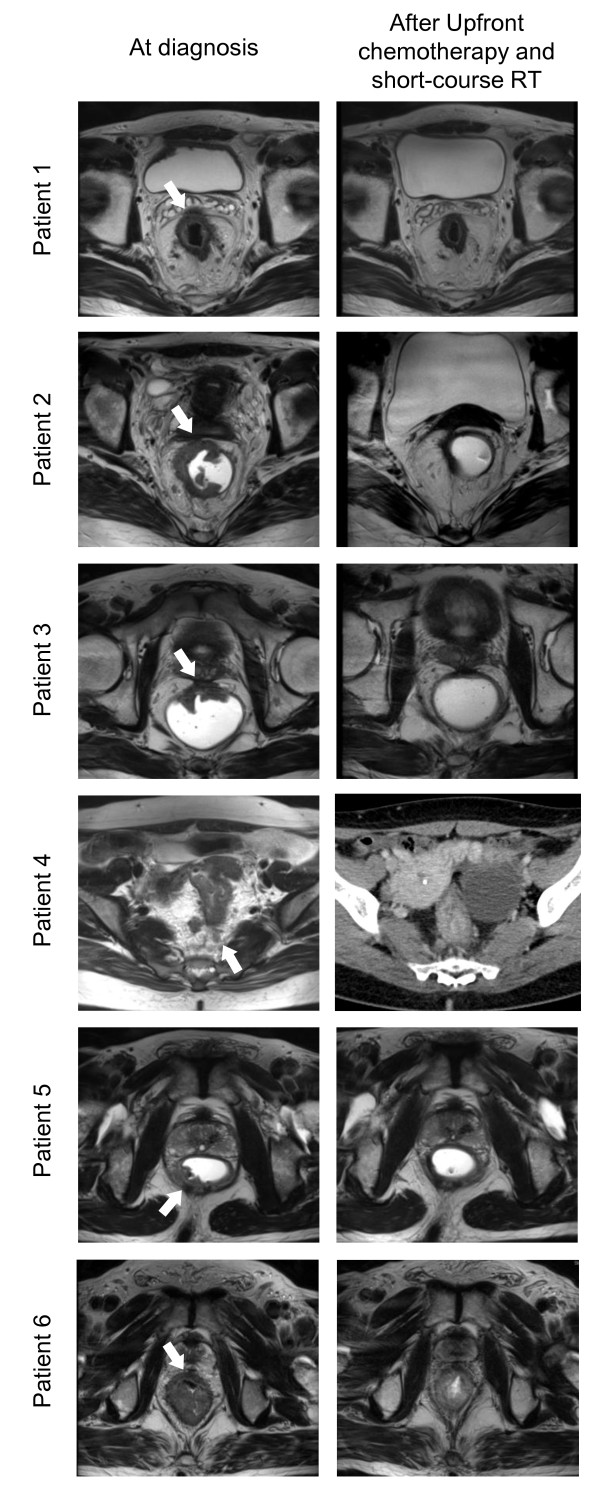
**MRI or CT at diagnosis and after upfront chemotherapy and short-course RT for each patient**. Arrow points to the mesorectal fascia involvement or closeness. After preoperative treatment, regression of rectal mass or lymph node was observed.

Most patients had a good clinical response based on imaging after pre-operative treatment. For primary rectal lesions, a low signal intensity change of the MRF involvement on MRI imaging was observed for patients 1, 2, 3, and 5. (Figure 1) Most metastatic lesions also regressed. Only one lesion, which was an ovarian metastasis in patient 4, progressed due to increased cystic fluid. All of the primary and metastatic lesions were resected simultaneously. The median duration between short-course RT and surgery was 13 weeks (range, 7-18 weeks). For primary rectal lesions, we performed low anterior resections (Table 2). For liver metastases, we performed lobectomy, wedge resection, or intraoperative radiofrequency ablation. Patient 2 underwent left oophorectomy for the left ovarian metastasis, and peritoneal washing cytology to detect peritoneal seeding. Peritoneal seeding masses were not observed during the operation. Bilateral salphingo-oophorectomy of both ovarian metastases was performed for Patient 4. Patient 6 showed pathologic complete remission of the primary rectal lesion. Complete TME of the primary rectal tumor was performed for all patients (Table 3). The tumor size was smaller than the MRI-predicted tumor size at the time of diagnosis for all patients. R0 resection was achieved in five patients (84%). Patient 5, who had a very low-lying tumor, had an R1 resection. There were no malignant cells in the peritoneal washing cytology and the left ovarian specimen from Patient 2. Metastatic adenocarcinoma was detected for all other metastatic lesions, which were completely resected.

**Table 2:** Surgery details and treatment outcomes after overall treatments

**Patient No**.	Interval from RT to OP (weeks)	Surgery	yp Stage*	Maintenance chemotherapy	Pattern of failure	Last follow up (months)	Survival
1	12	LAR, Lt. lobectomy and intraop RFA of liver	T3N1aM1a	FOLFOX #4	Distant at 15.7 months	23.5	AWD
2	8	LAR, Lt. oophorectomy	T3N0M0	A-FOLFOX #4	None	19.0	NED
3	14	LAR, Lt. lobectomy	T3N0M1a	None	None	16.9	NED
4	7	LAR, WR of Liver, BSO	T3N1aM1a	FOLFOX #11	None	16.5	NED
5	14	LAR, segmentectomy and WR of Liver	T3N2bM1a	FOLFOX #4	None	15.5	NED
6	18	LAR, WR of liver	T0N1aM1a	FOLFOX #4	None	15.8	NED

*Abbreviations: *RT, radiation therapy; OP, operation; LAR, low anterior resection; Lt., left; intraop, intraoperative; RFA, radiofrequency ablation; WR, wedge resection; BSO, Bilateral salphingo-oophorectomy; FOLFOX, 5-fluorouracil/leucovorin/oxaliplatin; A, avastin; AWD, alive with disease; NED, no evidence of disease.

*AJCC 7^th ^edition.

**Table 3:** Surgical pathologic reports

Patient **No**.	TME	Mandard grade	Tumor size	RM	Invasion depth	No. of LN dissected	No. of LN involved	LV invasion	Metastasis pathology
1	Complete	3	4	R0	Perirectal fat tissue	9	1	-	Liver; metastatic adenocarcinoma
2	Complete	2	0.7	R0	Perirectal fat tissue	18	0	-	Peritoneum; negative Lt. ovary; free from tumor
3	Complete	2	0.5	R0	Perirectal fat tissue	9	0	-	Liver; metastatic adenocarcinoma
4	Complete	3	2.3	R0	Perirectal fat tissue	28	2	-	Liver; metastatic adenocarcinoma both ovaries; metastatic adenocarcinoma
5	Complete	3	2.5	R1	Perirectal fat tissue	18	8	+	Liver; metastatic adenocarcinoma
6	Complete	1	0	R0	No tumor	22	1	-	Liver; metastatic adenocarcinoma

*Abbreviations: *TME, total mesorectal excision; RM, resection margin; LN, lymph nodes; LV, lymphovascular; Lt., left.

### Follow-up and toxicity

The median follow-up duration for all patients was 16.7 months (range, 15.5-23.5 months). The follow-up duration was defined as the interval between the date of diagnosis and the last follow up. There was no locoregional recurrence in any of the patients, but distant metastasis occurred in one patient. Patient 1 developed distant metastases in the liver and received salvage chemotherapy. Patient 1 survived with disease and all patients survived with no evidence of disease.

There were no severe acute toxicities within 1 week after short-course RT. During first chemotherapy after RT, three patients had grade 3 diarrhea. Between RT and surgery, 3 patients experienced acute grade 3 toxicities, which were controlled by conservative therapy. There were no other grade 3 or higher acute toxicity incidents. Five patients experienced acute grade 1 fatigue. Four patients experienced grade 1 anorexia. One patient experienced grade 1 diarrhea. A perirectal abscess was observed in Patient 1. He received abscess drainage and IV antibiotics, which resolved the perirectal abscess. No other surgical complications occurred during follow-up.

## Discussion

Optimal treatment strategies for patients with unresectable rectal cancer and synchronous systemic metastases are difficult to determine. Our retrospective study of six cases demonstrated that preoperative short-course RT with delayed surgery resulted in R0 resection for five patients with MRF involvement or closeness of rectal cancer and systemic metastases. In addition, upfront systemic chemotherapy controlled systemic metastases until metastatectomy.

In two European studies, preoperative short course RT (5 × 5 Gy schedule) with TME consistently improved the local control rate. A Dutch TME trial showed that the 5-year local recurrence rate was 10.9% for patients undergoing TME and 5.6% for patients undergoing preoperative RT [1,15]. In the Medical Research Council CR07, the 3-year local recurrence rates for patients undergoing TME or preoperative RT was 10.6% and 4.4%, respectively [2]. Currently, this short-course RT schedule with immediate surgery is a widely accepted standard treatment for rectal cancer. However, it is increasingly clear that MRF status, as determined by MRI scanning, has a substantial impact on the local recurrence rate and patient survival [7,8,16]. In the subgroup analysis of the Dutch trial, Marijnen *et al*. reported that patients with positive CRMs had a local recurrence rate of 17% and 30% after low anterior resection or abdominoperineal resection, respectively [11]. Unfortunately, postoperative treatment has not influenced both survival and local recurrence in the patients with CRM involvement. In the Medical Research Council CR07 trial, patients with positive CRMs who underwent postoperative chemoradiotherapy had a local recurrence rate of 11%, which did not compensate for positive CRMs like the subgroup analysis of the Dutch trial [2]. On the other hand, preoperative MRI accurately predicted MRF involvement or closeness in the MERCURY Study Group [8]. Therefore, when we identify a patient with either MRF involvement or closeness by preoperative MRI, we consider a treatment strategy that will induce macroscopic tumor regression and sterilization of surgical margin to achieve R0 resection.

Immediate surgery following short-course RT is not effective for tumor regression [12], whereas preoperative long course radiotherapy with chemotherapy can result in tumor down-staging [17]. Recently, a down-staging effect was documented after delayed surgery after short-course RT [14,18]. At Uppsala University, short course RT with delayed surgery was performed for 46 patients with had clinical non-resectable T4 disease with or without metastases [14]. Thirty-seven (80%) patients underwent surgery. R0 resection was achieved in 32 (86%) of these patients and a pathologic complete response was observed for four patients. Hatfield *et al*. treated 41 patients using short-course RT with delayed surgery [18]. MRI was used to determine the local tumor extent and its relationship with the MRF. Twenty-two (51%) patients had a MRF closeness (< 2 mm) and 20 (47%) had a MRF involvement. Of the 41 patients, 26 (63%) underwent surgical resection. Of the patients undergoing surgical resection, 22 (85%) had R0 resections, and two had pathological complete responses. These two retrospective studies show that short-course RT with delayed surgery can result in substantial down-staging for patients with either MRF involvement or closeness. In addition, R0 resection can be achieved for the majority of the patients treated with this regimen. Toxicity from short-course RT with delayed surgery was acceptable and comparable to long course RT with delayed surgery. Interim analysis of the Stockholm III trial demonstrated the feasibility, patient compliance, side-effects of RT, and early complications after surgery for different preoperative radiotherapy regimens (5 × 5 Gy and immediate surgery versus 5 × 5 Gy and delayed surgery versus conventional fractionated 50 Gy and delayed surgery). For patients treated with short-course RT and delayed surgery, severe acute toxicity was low (4.2%) and postoperative complications were not increased [19].

For metastatic disease, NCCN guidelines (v 2.2011) recommend that Initial treatment options for patients with stage IV disease with resectable liver or lung metastases include combination chemotherapy that has targeted agents, staged or synchronous resection of metastases, and rectal lesion or treatment with chemoradiotherapy. Upfront combination chemotherapy for the purpose of early eradication of micrometastases can be followed by staged or synchronous resection of metastases and rectal lesion, or by chemoradiotherapy for local control of disease prior to surgery. For the three groups of patients that received upfront chemotherapy, surgery should be delayed for 5-10 weeks following completion of such treatment [20]. However, the optimal sequence and timing of chemotherapy, RT, and surgery still remains controversial. For non-resectable primary rectal lesions with distant metastases, pelvic RT is needed to achieve down-staging and R0 resection, as well as local control of the rectal lesion prior to surgery. Simultaneously, systemic chemotherapy without dose reduction to control metastases is warranted. Upfront chemotherapy and short-course RT with delayed surgery is an attractive option in this clinical situation. Radu C *et al*. reported the results of treatment of 13 patients who had primary T4 tumors with synchronous distant metastases [14]. These patients were treated with systemic combination chemotherapy, integrating 5 × 5 Gy with delayed surgery. Surgery was performed for nine patients. R0 resection was achieved for six of the nine patients. Subsequent metastatic surgery was possible for two of the patients. In this study, six patients with MRF involvement or closeness and distant metastasis received similar sequences of chemotherapy, short-course RT, and delayed surgery. R0 resection of rectal lesions was possible for five patients. Furthermore, metastatic surgery was also successful in removing the tumor mass without evidence of microscopic disease. We totally agree with the suggestion of Radu and colleagues; Patients with MRF involvement or closeness of rectal cancer and synchronous distant metastases can be treated effectively with upfront systemic chemotherapy, short-course RT, delayed surgery, and systemic chemotherapy during the period of delay.

Our study has many limitations including small numbers, limited follow up period, heterogeneity of the combination of systemic agents and duration of treatments. Expansion to a larger study group is warranted. We are conducting a phase II clinical trial (NCT01269229), in which patients with MRF involvement or closeness of rectal cancer and liver metastases are treated with upfront systemic FOLFOX chemotherapy four cycles, 5 × 5 Gy RT to primary rectal lesion, repeat systemic FOLFOX chemotherapy four cycles, and delayed surgery [21].

## Conclusions

Upfront chemotherapy and short-course RT with delayed surgery appears to be a valuable alternative treatment for patients with MRF involvement or closeness of rectal cancer and distant metastases. The first advantage of this approach is that short-course RT with delayed surgery can result in down-staging and R0 resection for primary rectal lesions, which prevents local recurrence. The second advantage is that systemic chemotherapy without a dose reduction can result in early eradication of micrometastases and regression of metastatic tumor masses.

## List of abbreviations

RT: radiotherapy; TME: total mesorectal excision; MRF: mesorectal fascia; CRM: circumferential resection margin; MRI: magnetic resonance imaging; FOLFOX: 5-FU/leucovorin/oxaliplatin; CT: computed tomography; GTV: gross tumor volume; CTV: clinical target volume.

## Competing interests

The authors declare that they have no competing interests.

## Authors' contributions

NKK, SSJ and KWS carried out the conception, study design, acquisition of data, and data analyses and drafted the manuscript. HIY carried out data acquisition and data analysis. KYL, BSM, JBA, and KCK participated in data acquisition of, and revised the manuscript for intellectual content. All authors read and approved the final manuscript.
